# Unilateral mixed corticomedullary tumor of the adrenal gland with a synchronous contralateral pheochromocytoma: A case report and literature review

**DOI:** 10.1016/j.eucr.2026.103632

**Published:** 2026-10-01

**Authors:** Reza Kaffash Nayeri, Farshad Banouei, Maryam Abolhasani, Mohammad Mehdi Darzi

**Affiliations:** a Department of urology, Hasheminejad Kidney Center, school of medicine, Iran university of medical sciences, Tehran, Iran; b Fellowship in Molecular Pathology and Cytogenetic, Department of Pathology, Hasheminejad Kidney Center, School of Medicine, Iran University of Medical Sciences, Tehran, Iran

## Abstract

A 37-year-old man had two years of fatigue, weakness, and mood changes, initially misdiagnosed as depression. Workup showed hypertension, elevated urinary normetanephrine, elevated free cortisol, and suppressed ACTH. Imaging revealed bilateral adrenal masses (7.5 cm left, 4.7 cm right) with left adrenal vein thrombosis; MIBG scintigraphy showed bilateral uptake. After alpha-blockade, he underwent bilateral adrenalectomy. Inhibin positivity in the left mass's cortical region suggested a possible first-reported unilateral mixed corticomedullary tumor with synchronous contralateral pheochromocytoma, though entrapped cortical cells couldn't be excluded without further markers. Elevated Ki-67 and bilateral involvement warrant long-term follow-up and genetic evaluation.

## Highlights

• A 37-year-old man had fatigue, weakness, and mood changes for two years, initially misdiagnosed as depression.
• Bilateral adrenal masses caused dual hormonal secretion: elevated normetanephrine and cortisol with suppressed ACTH.
• Inhibin positivity suggested a possible unilateral mixed corticomedullary tumor with a contralateral pheochromocytoma.
• This combination has not been previously reported in the literature.
• Elevated Ki-67 and bilateral involvement warrant long-term follow-up and genetic evaluation.

## 1 Introduction

The adrenal gland consists of two parts with distinct embryological origins. The cortex arises from the mesoderm and produces steroid hormones, while the medulla originates from the neural crest ectoderm and produces catecholamines. Consequently, the presence of both cell types within a single tumor, known as a mixed corticomedullary tumor (MCMT), is a rare and unexpected occurrence.^1^ MCMT was first described by Mathison and Waterhouse in 1969, and fewer than 40 cases have been reported to date, making it one of the rarest adrenal tumors. Most patients are female, and the typical clinical presentation includes hypertension (69%) and Cushing syndrome (42%), reflecting the secretion of both cortisol (74%) and catecholamines (50%). This dual hormonal activity complicates preoperative diagnosis, which requires confirmation through histopathology and immunohistochemistry 1, 2, 3.

The pathogenesis of MCMT remains poorly understood. Tumor cells are distributed in a random, disorganized pattern, and one proposed mechanism involves aberrant differentiation of a pluripotent adrenal stem cell. Bilateral adrenal masses are uncommon and are more frequently associated with malignancy, tumor susceptibility syndromes, or hormonal disturbances.^4^^,^^5^ A unilateral MCMT occurring simultaneously with a contralateral pheochromocytoma has not been previously reported. We present such a case here, along with a review of the literature.

We searched PubMed, Scopus, and Embase from 1969 to July 2026 using the terms “mixed corticomedullary tumor” or “corticomedullary tumor” combined with “adrenal gland,” and manually screened the results for any mention of bilateral, contralateral, or synchronous pheochromocytoma. This search identified fewer than 40 reported unilateral MCMT cases and no previous reports of synchronous contralateral pheochromocytoma. Although we cannot completely exclude the possibility that a prior report exists but was indexed differently or used alternative terminology, literature reviews published alongside recent unilateral MCMT case reports also found no similar presentations,^6^^,^^7^ supporting the novelty of our case.

## 2 Case Presentation

The patient was a 37-year-old man with a two-year history of progressive fatigue, generalized weakness, lethargy, somnolence, and sleep disturbances. He had previously been referred to a psychiatrist due to mood changes and sleep problems and was diagnosed with depression. However, despite approximately one year of psychiatric treatment, his symptoms did not improve significantly.

On further questioning, he reported intermittent mild abdominal pain. There was no other relevant medical history. Physical examination revealed no pathological findings, and vital signs were within normal limits except for a blood pressure of 160/90 mmHg; heart rate and respiratory rate were normal. Abdominal and pelvic ultrasonography revealed two adrenal masses measuring 7.5 cm on the left side and 4.7 cm on the right side.

Further investigations were requested to determine the nature of these masses, and the results are presented in Table 1.

**Table 1:** Patient laboratory tests.

Blood Tests	Lab Test	Result	24 hours Urine Analysis	Lab Test	Result
	WBC	6700		Volume	2700 cc
	Hb	14.1 g/dl		Creatinine	1480 mg
	Urine Analysis	Normal		Metanephrine	190 μg
	Na	137 mEq/L		Normetanephrine	3600 μg
	K	4.2 mEq/L		Epinephrine	5.6 μg
	Ca	9.8 mg/dl		VMA	14.1 mg
	P	3.8 mg/dl		Free cortisol	> 200 μg
	PTH	17.5 pg/ml			
	Aldosterone	29.1 ng/dl			
	ACTH	5.6 pg/dl			

Contrast-enhanced abdominal and pelvic computed tomography (CT) revealed bilateral enlarging adrenal masses with thrombosis of the left adrenal vein (Fig. 1A and B). Due to these findings and the presence of biochemically active adrenal masses, the patient underwent an iodine-131 scan. The scan demonstrated two areas of abnormally increased radiotracer uptake in the upper abdomen, corresponding to both adrenal glands, with more intense uptake on the right side. The remainder of the body was unremarkable. These findings indicated two MIBG-avid foci in both adrenal glands, consistent with pheochromocytoma (Fig. 1C).

**Fig. 1:**
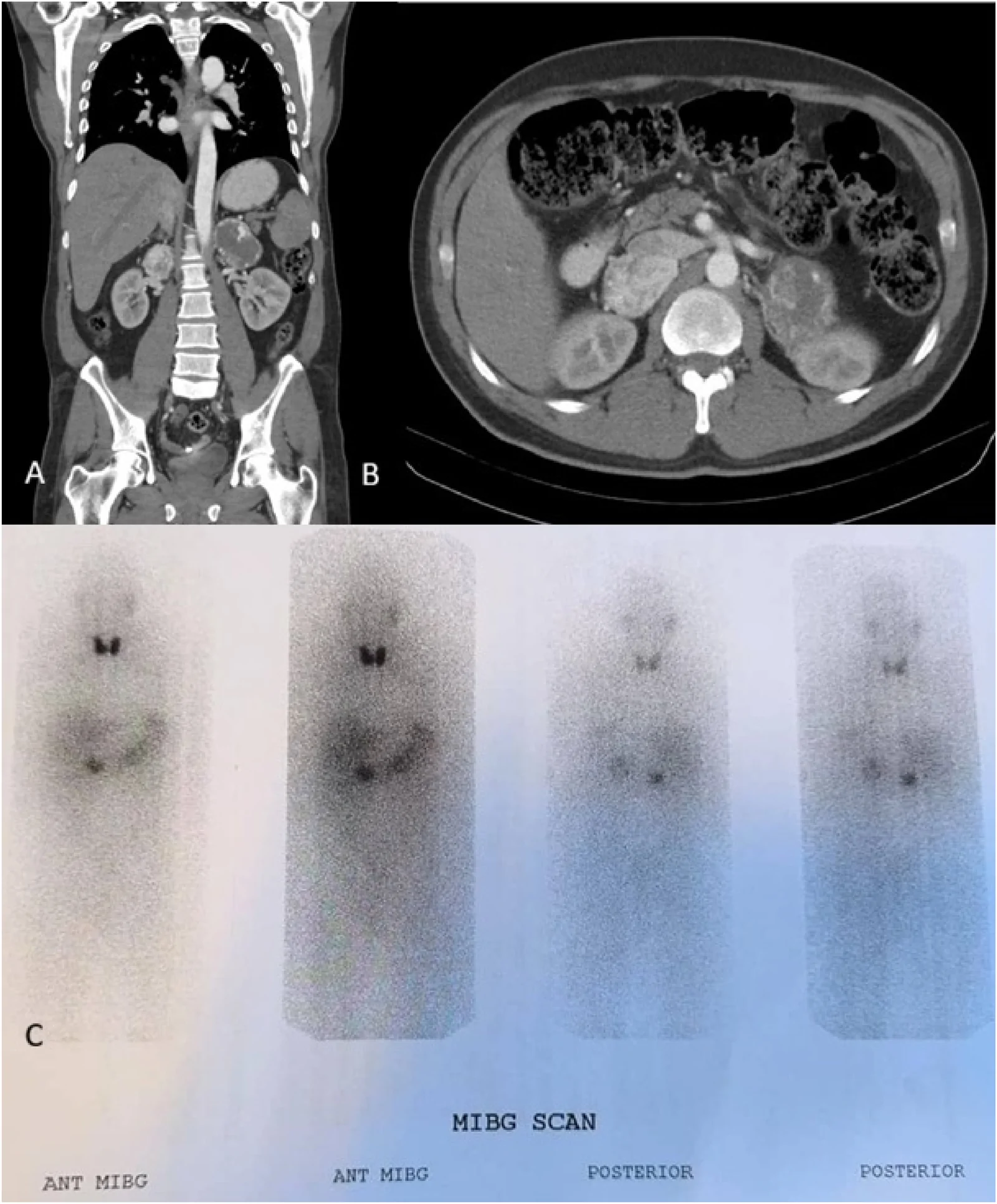
A & B: Computed tomography (CT) scan reveals large masses in both adrenal glands. C: Meta-iodobenzylguanidine (MIBG) scan reveals bilateral functional adrenal masses.

The patient was started on an adequate alpha-adrenergic blockade to prepare him for surgery. Bilateral adrenalectomy was then performed, and the resected specimens were sent for histopathological examination (Fig. 2, Fig. 3).

**Fig. 2:**
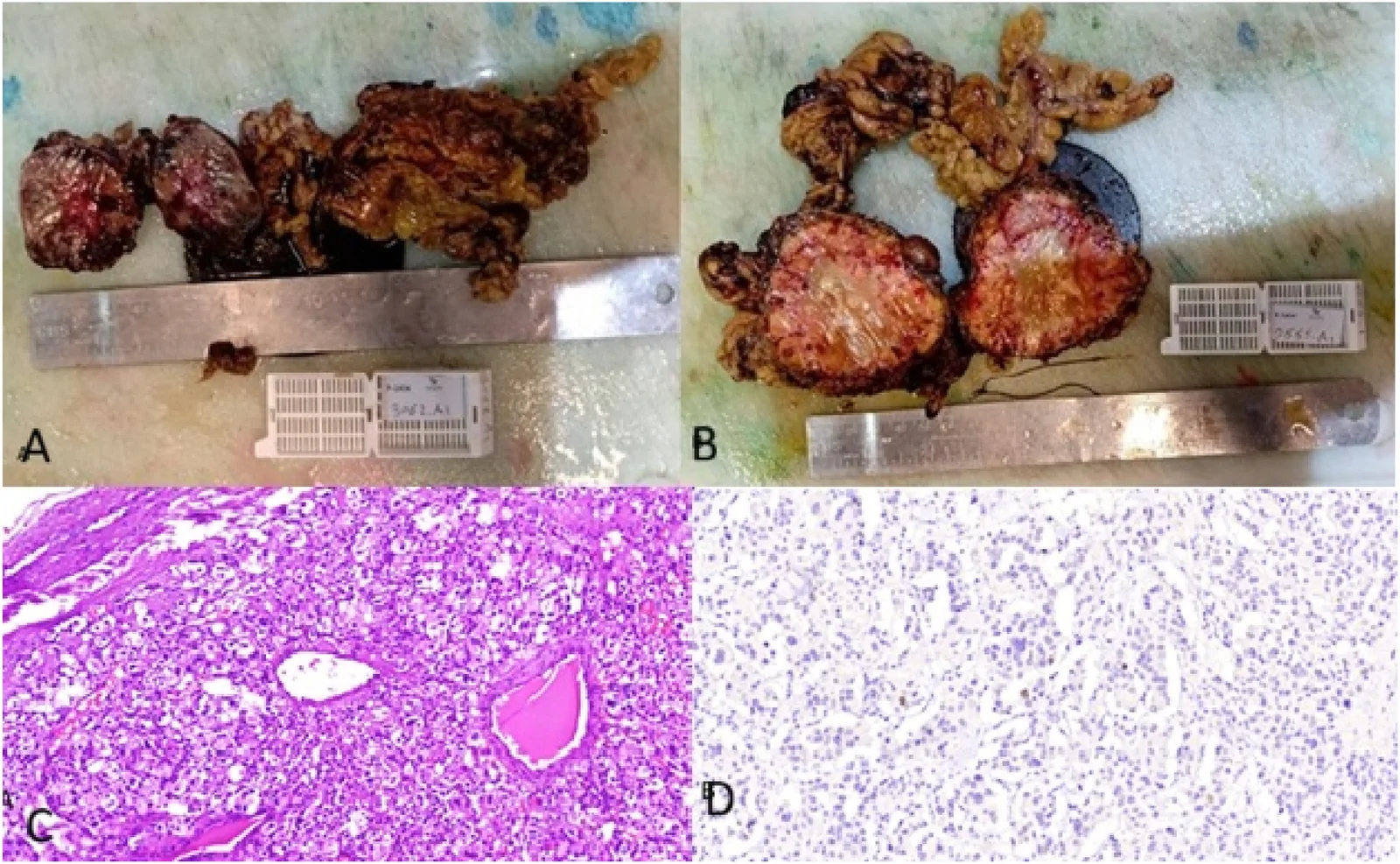
A: Macroscopy of the right adrenal mass showing a rubbery mass with a variegated creamy brown cut surface measuring 5 × 4.5 × 4 cm. B: Macroscopy of the left adrenal mass showing a rubbery mass with a variegated creamy orange cut surface measuring 8.5 × 7 × 5.5 cm. C: The H&E section of right adrenal mass shows zellballen nests of tumor cells without necrosis, capsular, sinusoidal or venous invasion in favor of benign behavior of tumor (x10). D. The proliferative index of Ki67 in right adrenal mass shows positivity only in about 3% of tumor cells in favor of benign behavior of tumor (x10).

**Fig. 3:**
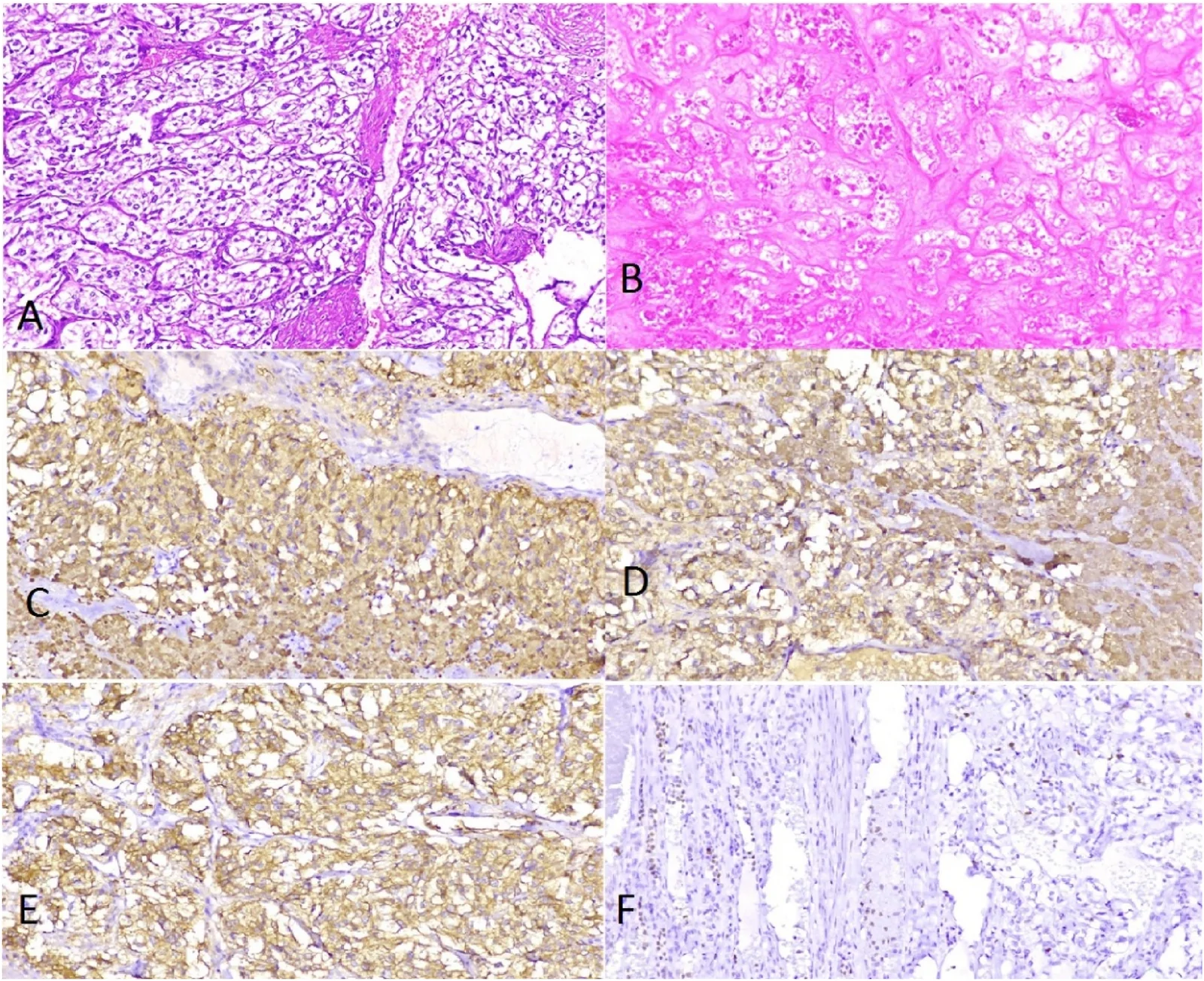
A: Microscopy of the left adrenal mass with zellballen nests of tumor cells with clear to eosinophilic cytoplasm and enlarged nuclei (×10). B: Microscopy of the left adrenal mass showing extensive foci of necrosis (×10). C: Immunohistochemistry (IHC) study shows cytoplasmic immunoreaction of synaptophysin in tumor cells (×10). D: IHC study shows cytoplasmic immunoreaction of chromogranin (×10). E: IHC study shows cytoplasmic immunoreaction of inhibin (×10). F: IHC study for Ki-67 shows positive immunoreaction in about 15% of tumor nuclei (×10).

The patient was discharged on postoperative day three in satisfactory general condition, on lifelong glucocorticoid (hydrocortisone) and mineralocorticoid (fludrocortisone) replacement therapy given the bilateral adrenalectomy, with endocrinology follow-up arranged for dose titration. During one year of follow-up, there was no evidence of disease recurrence, and tumor marker levels remained stable.

## 3 Discussion

Mixed corticomedullary tumors (MCMTs) of the adrenal gland are extremely rare neoplasms characterized by the coexistence of cortical and medullary cells within a single lesion, despite their distinct embryological origins.^1^ Our case is unique because it describes a unilateral MCMT occurring simultaneously with a contralateral pheochromocytoma, a presentation that has not been previously reported.^4^^,^^5^

In our patient, the two-year history of fatigue, weakness, somnolence, and mood disturbances was initially misdiagnosed as depression. This case illustrates the diagnostic challenges of MCMT, as its mixed hormonal activity often mimics psychiatric or endocrine disorders 1, 2, 3. Reported case series reveal a similar pattern, with hypertension being the most common presentation (69%), followed by Cushing syndrome (42%), and dual cortisol/catecholamine secretion observed in 74% and 50% of cases, respectively.

Laboratory tests revealed markedly elevated urinary normetanephrine and free cortisol levels, accompanied by suppressed ACTH, confirming autonomous, ACTH-independent secretion from both tumor components, consistent with previous reports.^1^^,^^3^ Serum creatinine was 1.1 mg/dL, within the normal range. The adequacy of the 24-h urine collection used for the catecholamine and cortisol measurements was supported by a urinary creatinine excretion of 1480 mg/24h, which falls within the expected range for an adult male and is consistent with a complete collection. CT imaging demonstrated bilateral adrenal masses measuring 7.5 cm on the left and 4.7 cm on the right, along with thrombosis of the left adrenal vein. On reflection, we no longer consider this thrombosis an incidental finding; given the subsequent histopathological demonstration of capsular, sinusoidal, and vascular invasion by the left adrenal mass, the venous thrombosis more likely reflects direct local tumor invasion of the adrenal vein rather than a separate, unrelated process. This is consistent with the aggressive local behavior otherwise documented in this tumor. Bilateral adrenal masses are uncommon and are frequently associated with malignancy, tumor susceptibility syndromes, or hormonal disturbances.^5^^,^^6^

The diagnosis was confirmed by histopathology. The left mass exhibited the typical zellballen pattern mixed with cortical elements, extensive necrosis, and capsular, sinusoidal, and vascular invasion 1, 2, 3. Immunohistochemistry (IHC) was positive for synaptophysin and chromogranin, which are medullary markers, as well as for inhibin, a cortical marker, suggesting differentiation of both lineages. Extended cortical markers such as SF-1 and Melan-A were not performed. Because inhibin can also stain entrapped non-neoplastic cortical cells, entrapment within an invasive pheochromocytoma cannot be excluded. Therefore, the diagnosis of MCMT in this mass should be considered provisional until confirmatory staining is completed. A Ki-67 index of approximately 15% indicated considerable proliferative activity, supporting the need for long-term surveillance. The right mass showed typical zellballen nests, positive for chromogranin and synaptophysin, with weak calretinin staining, no necrosis or invasion, and a Ki-67 index of about 3%, favoring a benign course. No clear neoplastic cortical component was identified in this mass, and extended cortical markers were not obtained (see Limitations). Based on the available evidence, the right mass is better classified as a pheochromocytoma, with the possibility of entrapped non-neoplastic cortical cells, rather than as a second MCMT.

The pathogenesis of MCMT remains poorly understood. The leading hypothesis suggests aberrant differentiation of a pluripotent adrenal stem cell capable of generating both lineages, supported by the disorganized mixing of cell populations observed across cases.^1^^,^^3^^,^^4^ The bilateral presentation raises the possibility of a germline tumor susceptibility syndrome; however, we found no features indicative of MEN2, VHL, SDH mutations, or NF1.

Management followed the standard protocol for functional bilateral tumors. The patient received preoperative alpha-blockade to prevent a hypertensive crisis,^1^^,^^2^ followed by bilateral total adrenalectomy, with an uneventful recovery. Cortical-sparing (subtotal) adrenalectomy, which can preserve adrenocortical function and avoid lifelong steroid dependence, was considered but not performed in this patient. This approach was felt to be unsuitable given the large size and necrosis of the left mass, its capsular, sinusoidal, and vascular invasion, and the diagnostic uncertainty regarding malignant potential on both sides; in this context, complete bilateral resection was prioritized over parenchymal preservation. Cortical-sparing surgery remains a reasonable alternative in patients with smaller, non-invasive bilateral tumors and should be discussed on a case-by-case basis. Lifelong glucocorticoid and mineralocorticoid replacement therapy was initiated.^5^ At the one-year follow-up, there was no recurrence; however, the elevated Ki-67 index in the left tumor indicates that ongoing surveillance remains necessary.^1^^,^^3^

Limitations: Germline genetic testing for MEN2, VHL, SDHx, and NF1 was not performed because there were no syndromic features present, and the test was unavailable at the time. Given the bilateral presentation and elevated Ki-67 index, genetic counseling and testing are recommended for future cases. Comprehensive hormonal profiling and additional immunohistochemical (IHC) markers, such as SF-1, Melan-A, and S100, were not obtained for either mass. The absence of SF-1 and Melan-A staining, along with the lack of a definitive neoplastic cortical component in the right mass, precludes a conclusive diagnosis of a second mixed corticomedullary tumor (MCMT) in that location. The same limitation applies to the left mass, where the MCMT diagnosis is based solely on inhibin positivity. However, inhibin cannot differentiate neoplastic cortical tissue from entrapped normal cortex, which is a critical consideration given the large size, necrosis, and invasion observed in this mass. If the cortical elements are determined to be non-neoplastic, the correct diagnosis would be bilateral pheochromocytomas rather than synchronous MCMT and pheochromocytoma, which would alter the primary claim of novelty in this report. Therefore, SF-1 and Melan-A staining are recommended, and until these results are available, the diagnosis should be considered provisional.

To our knowledge, this is the first reported case of unilateral MCMT occurring concurrently with a contralateral pheochromocytoma. This conclusion is based on inhibin positivity in the cortical elements of the left mass and should be considered provisional until confirmed with SF-1 or Melan-A staining, as bilateral pheochromocytomas remain a possible alternative diagnosis. With this caveat, the case contributes to the limited number of reports on this rare entity. Several practical points can be drawn from it. The nonspecific symptoms caused by dual hormonal secretion can lead to diagnostic delays, including misdiagnosis as a psychiatric condition. A comprehensive biochemical evaluation of both cortisol and catecholamine pathways is essential when bilateral adrenal masses are identified. MIBG scintigraphy can assist in confirming the diagnosis. A definitive diagnosis requires histopathology with a targeted immunohistochemical (IHC) panel that includes both medullary and cortical markers, including extended markers. The bilateral presentation and elevated Ki-67 index suggest that tumor susceptibility syndromes should be considered, and long-term oncologic surveillance is necessary. Further case reports and molecular characterization of future cases will be important.^1^^,^^3^

## CRediT authorship contribution statement

**Reza Kaffash Nayeri:** Conceptualization. **Farshad Banouei:** Writing – review & editing, Writing – original draft, Visualization, Validation, Supervision, Software, Resources, Project administration, Methodology, Investigation. **Maryam Abolhasani:** Data curation. **Mohammad Mehdi Darzi:** Supervision.

## IRB statement

This case report was reviewed and approved by the Institutional Review Board (IRB) of Hasheminejad Kidney Center, Iran University of Medical Sciences.

## Ethics approval and consent to participate

This case report was reviewed and approved by the Institutional Review Board (IRB) of Hasheminejad Kidney Center, Iran University of Medical Sciences.

## Consent for publication

Written informed consent was obtained from the patient for the publication of this case report and any accompanying images and associated clinical details.

## Availability of data and materials

The datasets generated and/or analyzed during the current case report are available from the corresponding author on reasonable request.

## Declaration of generative AI and AI-assisted technologies in the manuscript preparation process

During the preparation of this work, the author(s) used Claude (Anthropic, Claude Sonnet 5) in order to reduce word count to meet journal requirements and to revise the language of the Background, Case Presentation, and Discussion sections for clarity and readability. After using this tool, the author(s) reviewed and edited the content as needed and take full responsibility for the content of the published article. No scientific content, data, interpretation, or citation was added, altered, or removed beyond the original manuscript's meaning, and the AI tool had no influence on the study's conclusions, data analysis, or scientific interpretation.

## Funding

This research did not receive any specific grant from funding agencies in the public, commercial, or not-for-profit sectors.

## Declaration of competing interests

The authors declare that they have no known competing financial interests or personal relationships that could have appeared to influence the work reported in this paper.
